# Genotoxicity and cytotoxicity of the plasma jet-treated medium on lymphoblastoid WIL2-NS cell line using the cytokinesis block micronucleus cytome assay

**DOI:** 10.1038/s41598-017-03754-1

**Published:** 2017-06-20

**Authors:** Sung-Ha Hong, Endre J. Szili, Michael Fenech, Nishtha Gaur, Robert D. Short

**Affiliations:** 1 Future Industries Institute, University of South Australia, Adelaide, 5095 Australia; 2 Wound Management Innovation Cooperative Research Centre, Adelaide, 5000 Australia; 3 CSIRO Health Sciences and Nutrition, Adelaide, 5000 Australia; 4 Materials Science Institute, The University of Lancaster, City of Lancaster, LA1 4YW UK

## Abstract

Despite growing interest in the application of atmospheric plasma jets as medical treatment strategies, there has been comparatively little research on the potential genotoxic and cytotoxic effects of plasma jet treatment. In this study, we have employed the cytokinesis block micronucleus cytome (CBMN-Cyt) assay with WIL2-NS B lymphoblastoid cells to test the potential genotoxicity, as well as the cytotoxicity, of toxic species generated in cell culture media by an argon (Ar) plasma jet. Elevated levels of cell death (necrosis) and occurrence of chromosomal damage (micronuclei MN, nculeoplasmic bridge NPBs and nuclear bus, Nbuds) were observed when cells were exposed to plasma jet-treated media. These results provide a first insight into how we might measure the genotoxic and cytotoxic effect of plasma jet treatments (both indirect and direct) in dividing human cells.

## Introduction

Research into the applications of cold atmospheric plasma jets in biology and medicine has been rapidly growing in the past decade^1^. A wide range of plasma jet sources have been developed reflecting the growing commercial confidence in the likelihood of developing a new medical industry based on the use of plasma jets. This optimism is backed up by evidence in the selective destruction of cancerous cells^2^, the stimulation of wound healing^3^ and different dermatological applications^4, 5^.

In the recent years there has been a growing interest in the use of an indirect plasma treatment method, where plasma jet-treated media or plasma jet-treated liquid is used as a medical therapy. This method of treatment has been validated in several studies with success shown in the inactivation of gram-positive and negative strains of bacteria^6^, destruction of lung cancer cells^7^, brain tumour cells^8^, as well as destruction of *in vitro* 3D multicellular tumours^9^. It has been claimed that plasma jet-treated media has a certain degree of selectivity to cancerous cells, where breast cancer cells have been shown to be more susceptible to plasma jet-treated media than glioblastoma cells^10^. The indirect treatment method has also been shown to be effective in the destruction of chemo-resistant ovarian cancer cells^11^. In the indirect plasma treatment method, the composition of media is thought to be an important factor in generation of stable source of reactive species in treated media^12^ especially for treatment of cancer^13^.

To date, most researchers have attempted to link the medical benefits of plasma jets, operated with argon (Ar) or helium (He), to the reactive oxygen and nitrogen species (RONS) generated on interaction of plasma with the ambient air or liquid^14–16^. The interaction of ambient air treated by plasma jets with aqueous solution produces oxygen-containing species such as the hydroxyl radical (^**·**^OH), hydrogen peroxide (H_2_O_2_), superoxide (O_2_^−^) and peroxynitrite (ONOO^−^)^17, 18^. All of these species are likely to cause DNA abnormalities in cells^19–21^.

Given the growing interest in the biological and medical applications of plasma jets, it is essential to develop a detailed understanding of how to measure any potential genotoxic of plasma in human cells and follow the consequences of these in cell survival and cell division. Metrology of cell damage is needed to help mitigate potential safety concerns in the clinical use of plasma jets. A few studies in the literature have started to address this issue including studies of changes in gene expression following exposure to plasma treated media^22^ and single/double strand breaks in naked DNA^23–25^. Relevant to our study, Wende *et al*. have monitored the genotoxicity of plasma in cell culture by counting the formation of micronuclei (MN)^26^. A further study has used the formation of MN to follow damage in the dielectric barrier discharge plasma treatment of brain cancer cells - showing that the increased frequency of MN correlated with the plasma jet exposure time^27^. The cytotoxicity of direct and indirect plasma treatment is more typically studied, using various assays such as viability assays using resazurin^18^ or MTT^10^ and fluorescence staining methods to track and visualise live/dead cells^28^ routinely employed.

The cytokinesis-block micronucleus cytome assay (CBMN-Cyt) is a method which allows the investigation of both genotoxicity as well as cytotoxicity and has great versatility in the number of different measurements that can be made within one single experiment^29^. A previous study verified the suitability and sensitivity of the CBMN-Cyt to investigate DNA damage and cell death in lymphoblastoid cells induced by H_2_O_2_ and O_2_^−^^30^. The CBMN-Cyt assay has been validated in independent systems to examine genetic alterations caused by ionizing irradiation^31^, chemical carcinogens^32, 33^, nutrient deficiency^34^ and heavy metals^29^. The CBMN-Cyt assay has been endorsed by the OECD as a required test for determining genotoxicity of chemicals^35^ and by the IAEA for biodosimetry of ionizing radiation exposure^36^. The protocol we have followed employs the WIL2-NS B lymphoblastoid cell line. This cell is particularly relevant to plasma medicine in the context of treating inflamed or infected sites, as it is typical of the cells involved in the immune response^37–39^, thus it is one of the most prominent cells found at these sites. Furthermore, the genotoxicity and cytotoxicity for monitoring other medically relevant technique such as ionizing radiation^40^ and chemotherapy^41^ have been studied extensively using the CBMN assay and the WIL2-NS cell line.

In this study, we investigated the effects of an Ar plasma jet-treated Rosewell Park Memorial Institute 1640 cell culture medium (herein referred to as RPMI 1640) on WIL2-NS cells originally isolated from the spleen of a Caucasian male^42^, using the CBMN-Cyt assay. We investigated the genotoxicity through scoring the occurrence of various chromosomal damage biomarkers. We also measured cytotoxicity through the measure of apoptotic and necrotic cells, as well as through a cell viability study. Finally, we quantified the concentration of H_2_O_2_ generated in RPMI 1640 following plasma jet treatment and proposed a link between the genotoxicity of plasma-jet treated media and the generation of H_2_O_2_.

## Results

### Genotoxicity of Ar plasma jet-treated RPMI 1640

**Figure 1:**
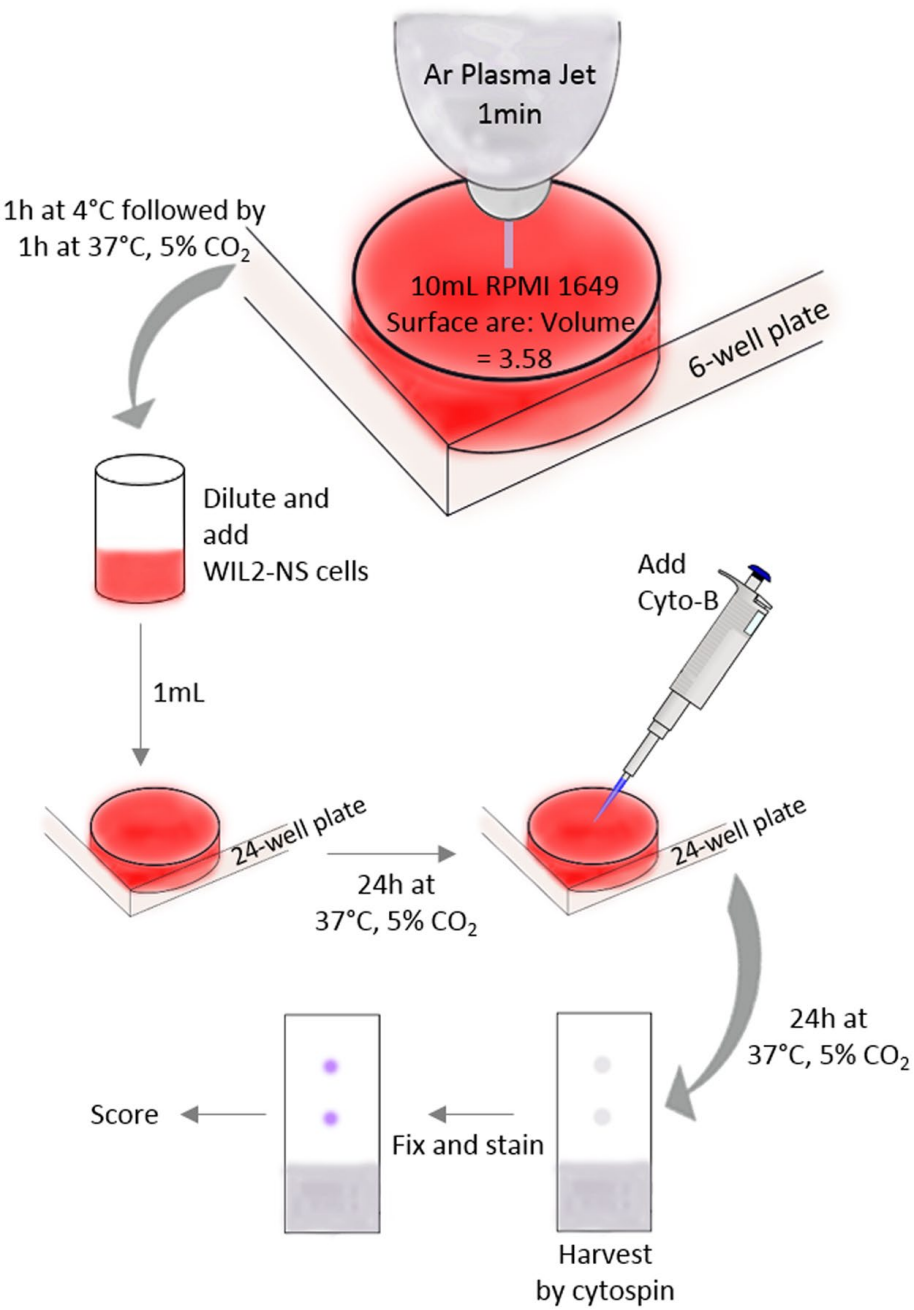
Protocol used to assess the genotoxicity and cytotoxicity of Ar plasma jet-treated RPMI 1640.

The experimental procedure from plasma treatment of RPMI 1640 through to the steps taken to perform the CBMN-Cyt assay are shown in Fig. 1. A detailed description of the CBMN-Cyt (including scoring criteria) has been published in^43^. In brief, RPMI 1640 was treated with an Ar plasma jet, the media was then transported at 4 °C to the facility where the CBMN-Cyt assay was performed. The treated media was then incubated for 1 h at 37 °C before media supplements were added and cell suspensions were exposed to treated media. The WIL2-NS cell line was chosen for this study, as this cell line has been validated for use in the CBMN-Cyt assay in a number of previous studies^29, 30, 34^. It is suited to this type of damage identification due its high nuclear division index and p53 deficiency, which allows cells with DNA damage to survive - facilitating DNA damage “events” to be observed and quantified^30^.

**Figure 2:**
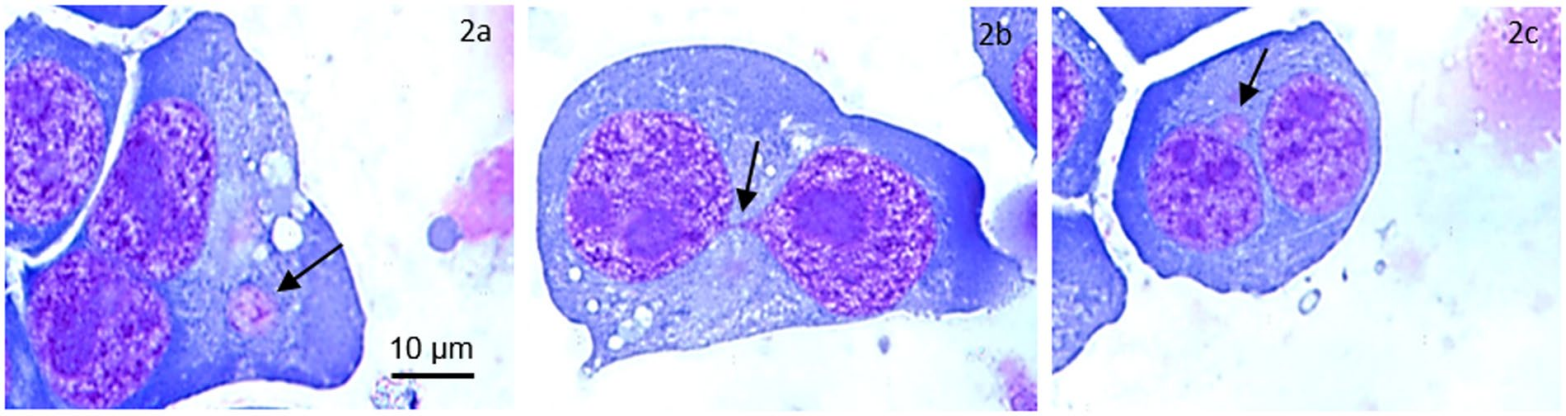
Binucleated WIL2-NS cells following incubation in Ar plasma jet-treated RPMI 1640 with (**a**) a micronucleus (MN), (**b**) a nucleoplasmic bridge (NPB) and (**c**) a nuclear bud (Nbud). Arrows indicate the corresponding biomarkers. Magnification ×1000.

The CBMN-Cyt assay is based upon scoring chromosomal damage events, which are expressed as nuclear anomalies in once-divided cells which are accumulated and identified as binucleated (BN). The recognized biomarkers for chromosomal damage in the CBMN-Cyt assay are MN, nucleoplasmic bridges (NPBs) and nuclear buds (Nbuds)^44^. Figure 2 shows microscopic images of these biomarkers. MN are formed through chromosomal fragments or whole chromosomes, which lag behind during anaphase in mitosis. NPBs arise from dicentric chromosomes that are pulled to opposite poles of the cell thus forming a dicentric chromosome bridge between nuclei of a BN cell and are indicative of misrepair of DNA breaks or telomere and fusion^30^. Nbuds have recently been established as a biomarker of chromosomal instability, and are thought to be caused by exclusion of excess amplified genetic material^45^. Scoring of these biomarkers is strictly restricted to once-divided BN cells and for this reason this assay uses cyto-B to block the cell division cycle at the stage of cytokinesis.

**Figure 3:**
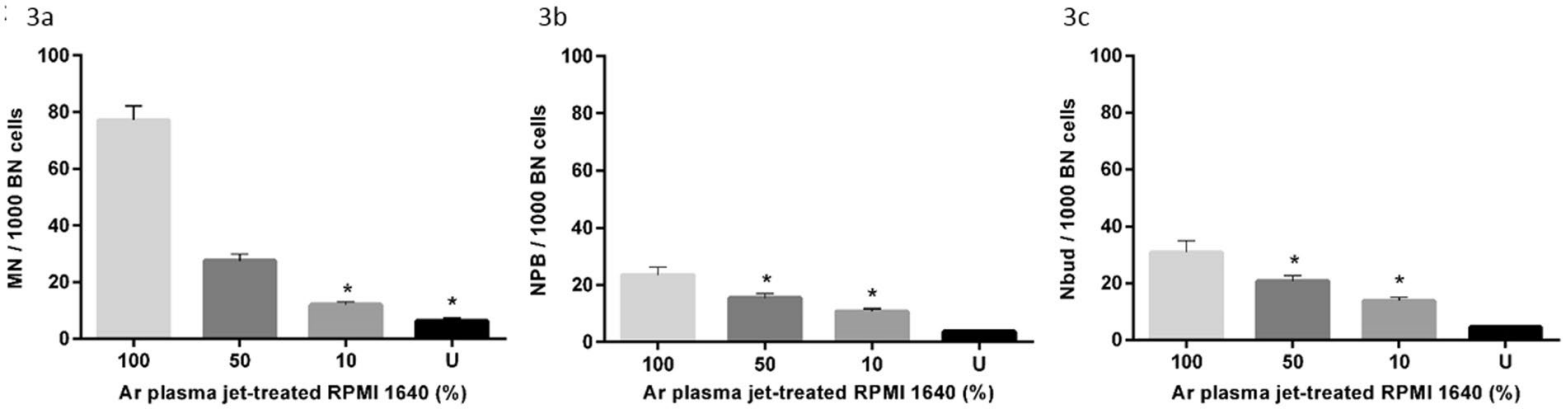
Chromosomal damage in untreated (U) RPMI 1640 and in Ar plasma jet-treated RPMI 1640 (at concentrations of 10, 50 and 100%). Damage was scored in WIL2-NS cells using the CBMN-cyt assay: (**a**) frequency of MN, (**b**) frequency of NPBs, and (**c**) frequency of Nbuds per 1000 binucleated cells. Experiments were performed in triplicate with triplicate samples per experiment (*n* = 9). Each point represents the mean value and ± standard error of the mean (SEM) of all repeats. One-way ANOVA was used to determine the statistical significance of the values. The values without a mark above indicate that they are statistically significantly different whereas the values sharing * are not (*P* < 0.05).

Figure 3 shows the frequency of chromosomal damage (MN, NPB, Nbud) scored after incubation of cells in Ar plasma jet-treated RPMI 1640. Figure 3a shows a higher frequency of MN cells in the undiluted and less dilute Ar plasma jet-treated RPMI 1640. Cells cultured in the undiluted (100%) Ar plasma jet-treated RPMI 1640 showed approximately a 2-fold increase in the frequency of MN compared to cells cultured in 50% Ar plasma jet-treated RPMI 1640, and approximately a 10-fold increase compared to untreated RPMI 1640. The values were shown to be significantly different with one-way ANOVA (*P* < 0.05). Similar trends were observed in the other biomarkers of chromosomal damage, i.e. NPB (Fig. 3b) and Nbuds (Fig. 3c), with the highest level of damage observed when exposed to undiluted Ar plasma jet-treated RPMI 1640 (*P* < 0.05).

### Cytotoxicity of Ar plasma jet-treated RPMI 1640

**Figure 4:**
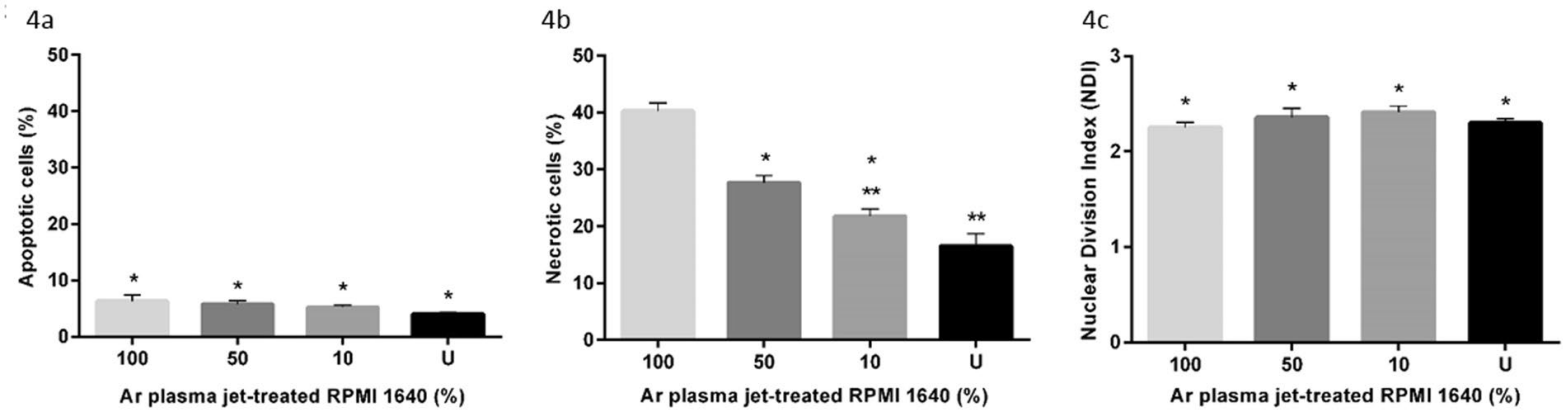
Cytotoxicity of untreated (U) RPMI 1640 and Ar plasma jet-treated RPMI 1640 (at concentrations of 10, 50 and 100%): (**a**) percentage of apoptotic cells, (**b**) percentage of necrotic cells, and (**c**) Nuclear Division Cytotoxic Index (NDI). Experiments were performed in triplicate with triplicate samples per experiment (*n* = 9). Each point represents the mean value and ± standard error of the mean (SEM) of all repeats. One-way ANOVA was used to determine the statistical significance of the values. The values without a mark above indicate that they are statistically significantly different whereas the values sharing * or ** are not (*P* < 0.05).

Cytotoxicity was also assessed by scoring the frequency of apoptotic and necrotic cells, which were expressed as a percentage of the total cell population (Fig. 4). The occurrence of apoptotic (Fig. 4a) and necrotic (Fig. 4b) cells was greater in the 100% and less diluted Ar plasma jet-treated RPMI 1640; but was only significant for necrotic cells (*P* < 0.05) but not for apoptotic cells (*P* > 0.05). It is important establish whether the plasma treated jet media had any effect on the rate of cell division. This was measured through the nuclear division index (NDI), which was found to be similar (at c*a*. 2) for all dilutions of Ar plasma jet-treated RPMI 1640 (Fig. 4c). This value is what would be expected for a cell with a doubling time of ca. 48 h.

**Figure 5:**
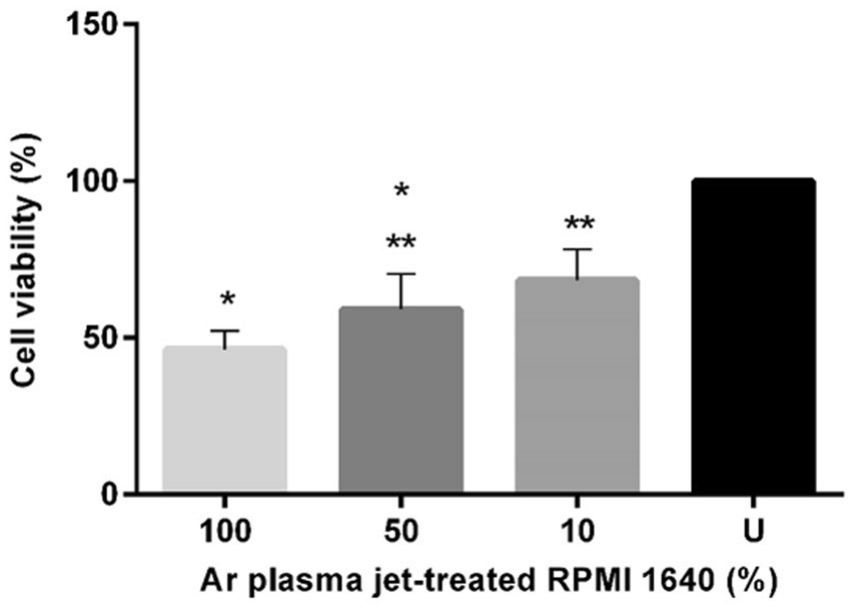
Percent cell viability (determined with the resazurin assay) after 48 h incubation in untreated (U) RPMI 1640 and Ar plasma jet-treated RPMI 1640 (at concentrations of 10, 50 and 100%). Each point represents the mean value and ± standard error of the mean (SEM) of samples analyzed in triplicate (*n* = 3). One-way ANOVA was used to determine the statistical significance of the values. The values without a mark above indicate that they are significantly different whereas the values sharing * or ** are not (*P* < 0.05).

Figure 5 shows that cell viability, measured using a resazurin cell viability assay, decreased for cells cultured in the less dilute Ar plasma jet-treated RPMI 1640, with viability of cells exposed to undiluted Ar plasma jet-treated RPMI 1640 being significantly lower than cell viability exposed to untreated RPMI 1640 (*P* < 0.05).

### Concentration of Hydrogen peroxide generated in RPMI 1640 by Ar plasma jet treatment

Of the RONS known to be generated by plasma-jets in solutions, H_2_O_2_ is a relatively stable product (especially in the absence of FBS)^13^ which can be detected days after plasma exposure^46^. Using a “Free Radical Analyzer” (WPI Instruments), changes in H_2_O_2_ concentration in RPMI 1640 were measured immediately after Ar plasma jet treatment and following 2 h incubation (the time-point at which the Ar plasma jet-treated RPMI 1640 was applied to cells as shown in Table 1). Immediately after Ar plasma jet treatment of RPMI 1640, a H_2_O_2_ concentration of 25 µM was detected before dilution (*i.e*. at 100%); and the concentration of H_2_O_2_ decreased with increased dilution (*P* < 0.05). The media was then incubated at 4 °C, which was previously reported to be within the ideal storage temperature to retain activity of plasma treated media^9^, for transport between laboratories. Then the media supplements such as FBS were added and the media was further incubated at tissue culture conditions (37 °C and 5% CO_2_). Following the 2 h incubation, the mean H_2_O_2_ concentration was reduced to 18.3 µM before any dilution (100%) and 12.9 µM for a 50% dilution. This reduction in concentration of H_2_O_2_ was expected as addition of supplements such as FBS containing proteins, have been shown to interfere with the stability of H_2_O_2_^10, 12^.

**Table 1:** Concentration of H_2_O_2_ in untreated (U) RPMI 1640 and Ar plasma jet-treated RPMI 1640 (at concentrations of 10, 50 and 100%).

Concentration of plasma treated media (%)	*H*_*2*_ *O*_*2*_ *concentration following incubation (µM)*	
	*0* *h*	*2* *h*
U	…	…
10	2.76 ± 0.09	…
50	12.39 ± 0.09	12.9 ± 3.06
100	25.41 ± 0.73	18.3 ± 4.08

Measurements were taken immediately following Ar plasma jet treatment (0 h) and following 2 h incubation (time-point of application to cells). The data presented is the mean value and ± standard error of the mean (SEM) of samples analyzed in triplicate (*n* = 3). One-way ANOVA was used to determine the statistical significance of the values. All values are statistically significantly different (*P* < 0.05).

…Indicates concentration below detection limit.

## Discussion

The purpose in undertaking this study was to explore the versatility of the CBMN-Cyt assay for measuring whole cell and chromosomal responses to plasma jet-treated media and later direct plasma. The study was not undertaken to assess the safety of plasma *per se*. In this respect a number of key results were observed with the WIL2-NS lymphoblastoid cell line - these include, there was no discernible effect on the rate of cell division (as measured through the NDI) but an increased cell death (necrosis, but not apoptosis) and a strong display of a range of markers of abnormal cell division – MN, NPBs and Nbuds. Cell death and raised markers of chromosomal damage were greatest in the undiluted and less diluted plasma jet-treated media. The WIL2-NS cell is p53 deficient and therefore we would not have expected apoptosis. WIL2-NS cells have a fast rate of division and are more likely to propagate abnormalities in cell division. And in this context our data are different to those of Wende *et al*.^26^ who performed a micronuclei (MN) assay count with HacaT cells (keratinocytes) exposed to plasma-treated media. Wende *et al*., based on MN counts, reported no effect of plasma treatment on the cell division process. This difference is not at all surprising as the full CBMN-Cyt assay has revealed markedly different responses in different cell types to the same agents^47^. Furthermore, the full CBMN-Cyt assay using lymphoblastoid cell line has been previously validated for measuring NPBs, a marker used as an indication of chromosome loss and breakage and Nbuds, a marker used as an indication of over amplification of gene. This allows for a much more detailed assessment of toxicity compared to the simple measure of MN^48^.

Of the likely longer lived RONS in the plasma jet-treated media our suspicion falls upon H_2_O_2_ as the likely agent of the effects we report. The dose dependent toxicity of H_2_O_2_ in various (and different) cell lines has been established in previous studies (of particular relevance is Umegaki *et al*.^30^, which is discussed below). Available data indicate that even small doses of H_2_O_2_, as low as 10 µM, can be cytotoxic and genotoxic, and may even be related to development of cancer^49^. Low doses of RONS produced by neutrophils in the inflammation process can induce oxidative damage in the DNA of normal cells^50^. It has been shown that H_2_O_2_ can easily penetrate across the phospholipid membrane of cells utilising water channels such as aquaporins^51, 52^. Once in the cellular interior, H_2_O_2_ poses a particular threat in the nucleus, where DNA damage results from the generation of OH radicals^53^ (OH radicals are known to be generated from H_2_O_2_ by Fe^2+^ ions within cellular compartments through the Fenton reaction)^54^. OH radicals attack DNA at the sugar residues of the DNA backbone, which results in single-strand breaks^53^. Without repair, these single-strand breaks may be carried through to double-strand breaks, which are expressed as chromatid breaks, *i.e*. a potential pathway to the formation of MN^55^.

The study of Umegaki *et al*. provides an optimal control for our study. In their paper, Umegaki *et al*. have exposed WIL2 NS cells to varying concentration of H_2_O_2_. Our current study and that in ref. 30 were performed within the same laboratory, using the same methods, cell line and equipment. As in this current study, Umegaki *et al*. used the CBMN-Cyt assay to assess the genotoxicity in WIL2-NS cells after incubation of these cells. The physiologically relevant concentrations of H_2_O_2_ (concentrations of H_2_O_2_ produced by activated neutrophils during the process of immune response) used by Umegaki *et al*. correlates well the concentration of range of H_2_O_2_ generated by the Ar plasma jet in RPMI 1640.

**Figure 6:**
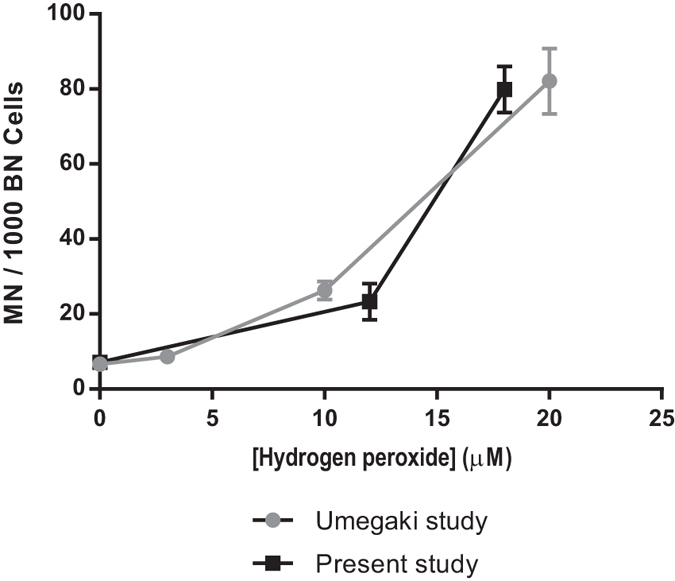
MN frequency in WIL2-NS cells as function of H_2_O_2_ concentration. The trend in MN formation versus H_2_O_2_ concentration was compared between the data presented by Umegaki and Fenech (Umegaki study)^30^ here H_2_O_2_ was added as a solution, to the data in this paper (Present study), where H_2_O_2_ was added by an Ar plasma jet. For “Umegaki study” the data points were obtained from triplicate measurements (*n* = 3)^30^ for “Present study” the data points were obtained from triplicate experiments with triplicate samples per experiment (*n* = 9). Each point represents the mean value and ± standard error of the mean (SEM).

Therefore to compare our results with those in Umegaki *et al*., the level of DNA damage (expressed as MN) is plotted as function of H_2_O_2_ concentrations in Fig. 6. From this figure we can see that the level of DNA damage is comparable at the equivalent H_2_O_2_ concentrations. This indicates that of all the RONS generated by the Ar plasma jet in RPMI 164, H_2_O_2_ is likely to be the major contender in inducing DNA abnormalities in WIL2-NS cells.

Some of the other potential effects of plasma have been investigated. Beyond generating RONS, we found that Ar plasma jet treatment did not result in any change in pH or any temporary elevation of the temperature of the RPMI 1640. However, the potential modification of micronutrients and proteins was not analysed. Such modification of micronutrients and proteins by the Ar plasma jet would result in a decrease their bioavailability and bioefficacy, and a nutritional deficiency, which has been linked to chromosomal damage^56^.

The direct extrapolation of the data presented in this paper to the likely effects in treatment of real tissue (and on lymphocytes *in situ* of, for example, a wound) should be approached with caution. Significant differences are, for example the static and 2D nature of *in vitro* cell culture versus real tissues which are 3D and would be subject to constant flow of fluid. The latter would continuously replenish the treatment site, removing RONS and replacing denatured biomolecules with fresh micronutrients and proteins and removing toxic waste generated by the plasma jet treatment. Interstitial fluid and blood plasma is also complete with endogenous antioxidants such as catalase and glutathione peroxidase^57^. In this study, the effects of antioxidants were not considered, but would be expected to reduce the accumulation of RONS at the treatment site. Furthermore, the intracellular concentration of RONS was not monitored in this paper. Monitoring intracellular RONS using RONS scavengers such as NAC^58^ will provide indications for understanding the mechanism of genetic damage caused by plasma jet treatment. Therefore, in the direct or indirect exposure of plasma or medium pre-treated with plasma to living tissue from animal or human subjects, dynamic changes in the concentrations of RONS should be carefully considered, as recently discussed^59, 60^.

In conclusion, the CBMN-Cyt assay with WIL2-NS cells is an effective system to detect whole cell and chromosomal damage induced by Ar plasma jet-treated cell culture media. Trends in the extent of DNA damage mirror the cytotoxic (necrotic) effect of Ar plasma jet-treated cell culture media. These results highlight the possible survival of genomically-abnormal cells. And whilst this observation may warrant careful consideration in the context of potential genetic aberrations in the *in situ* medical use of plasma jets, our objective in these preliminary experiments was to highlight the ***full*** potential of the CBMN-Cyt assay as a tool for assessing the cytotoxicity and genotoxicity of cold atmospheric plasmas. Such methods will become increasingly important in the development of plasma technology for the targeted and controlled destruction of cancer cells, by demonstrating targeted DNA damage and destruction in cancers, whilst in normal (adjacent) tissue the minimization of DNA damage and cell death.

## Materials and Methods

### Ar plasma jet treatment of RPMI 1640

A commercial Ar plasma jet was operated at 1 MHz with an Ar gas flow rate of 5 L/mins. The Ar was ultra-high purity grade (BOC). A volume of 10 mL of RPMI 1640 supplemented with 5% (v/v) foetal bovine serum (Sigma) was treated in 6-well tissue culture polystyrene (TCPS) plates at a distance of 20 mm between the end of the nozzle of the plasma jet assembly and the top of the plate.

### Exposure of cells to Ar plasma jet-treated RPMI 1640

After Ar plasma jet treatment, the RPMI 1640 was transported cold to the cell culture facility, which took approximately 1 h on ice, thus there were incubated at 4 °C for 1 h. The Ar plasma jet-treated RPMI 1640 was supplemented with 100 IU/mL penicillin and 100 mg/mL streptomycin solution and 1% L-glutamine (Sigma), then incubated for 1 h at 37 °C in a humidified incubator with 5% CO_2_, this was to bring the cell culture medium to optimum condition before exposure to cells. Therefore, there was an unavoidable delay of 2 h before 5 mL of serial dilutions of the Ar plasma jet-treated RPMI 1640 were prepared and 50 µL of cell suspension was added to obtain an end cell count of 2 × 10^6^ viable cells/mL. A 1 mL volume of the cell suspension, prepared in RPMI 1640, or undiluted or diluted Ar plasma jet-treated RPMI 1640, was distributed into each well of a 24-well TCPS plate. Cells were exposed to Ar plasma jet-treated RPMI 1640 used either undiluted or diluted to 50% or 10% (with dilutions prepared in pre-warmed, untreated RPMI 1640). The cells were incubated for 24 h under the cell culture conditions described above.

### CBMN–Cyt assay

After incubation of cells in Ar plasma jet-treated RMPI 1640, the cytochalasin-B (Cyto-B) (Sigma) was added and the cells were incubated for a further 24 h before being harvested onto glass slides using a “cytospin”, air dried for 10 min, and fixed and stained using “Diff-Quick” for scoring. Figure 2 shows the process of Ar plasma jet treatment of RPMI 1640 through to preparation of microscope slides for scoring. A total of 1000 BN cells were scored for the presence of MN (Fig. 2a), NPBs (Fig. 2b) and Nbuds (Fig. 2c) per culture condition following an established protocol^31^. Chromosomal damage was scored and expressed as the number of damage events per 1000 BN cells. A total of 250 cells, including mononucleated, BN, multinucleated, apoptotic and necrotic, were counted per slide. The data from these cell counts were used to determine the cytotoxicity (only apoptotic and necrotic cells), as well as the nuclear division index (NDI) for all cells. The NDI is a marker used for nuclear division status and can be used as a marker to determine the cytostatic effects of the Ar plasma jet used in this study. The methodology used to score and calculate cytotoxicity, NDI and DNA damage biomarkers, was previously described^55^.

### Resazurin assay

Cytotoxicity assessment of cells exposed to diluted Ar plasma jet-treated RPMI 1640 was also carried out using a resazurin cell viability assay^61^. After 24 h incubation of cells in Ar plasma jet-treated RPMI 1640, 100 µl of 100 µg/mL resazurin (Sigma) prepared in RPMI 1640 was added and incubated for 24 h. Following incubation, cell suspensions in the 24-well TCPS plate were centrifuged to remove cells. The fluorescence intensity of the supernatant was measured at an excitation and emission wavelength of 560 nm and 590 nm, respectively. The fluorescence intensity (FI) of cells exposed to Ar plasma jet-treated RPMI 1640 was normalised against FI of cells exposed to untreated RPMI 1640 following the equation: [(FI treated cells − FI untreated cells)/FI untreated cells] ×100%.

## References

[1] Von Woedtke T, Metelmann HR, Weltmann KD (2014). Clinical plasma medicine: state and perspectives of *in vivo* application of cold atmospheric plasma. Contrib. Plasm. Phys..

[2] Keidar M (2011). Cold plasma selectivity and the possibility of a paradigm shift in cancer therapy. Br. J. Cancer, BJC.

[3] Isbary G (2012). Successful and safe use of 2 min cold atmospheric argon plasma in chronic wounds: results of a randomized controlled trial. Br. J. Dermatol..

[4] Heinlin J (2013). Randomized placebo‐controlled human pilot study of cold atmospheric argon plasma on skin graft donor sites. Wound Repair Regen..

[5] Weltmann KD (2012). Plasma processes and plasma sources in medicine. Contrib. Plasm. Phys..

[6] Kamgang‐Youbi G (2009). Microbial inactivation using plasma‐activated water obtained by gliding electric discharges. Lett. Appl. Microbiol..

[7] Adachi T (2015). Plasma-activated medium induces A549 cell injury via a spiral apoptotic cascade involving the mitochondrial–nuclear network. ‎Free Radic. Biol. Med.

[8] Tanaka, H. *et al*. Plasma-activated medium selectively kills glioblastoma brain tumor cells by down-regulating a survival signaling molecule, AKT kinase. *Plasma Medicine* **1** (2011).

[9] Judée, F. *et al*. Short and long time effects of low temperature Plasma Activated Media on 3D multicellular tumor spheroids. *Sci. Rep*. **6** (2016). 10.1038/srep21421 PMC4761900 26898904

[10] Yan D (2015). Principles of using cold atmospheric plasma stimulated media for cancer treatment. Sci. Rep..

[11] Utsumi F (2013). Effect of indirect nonequilibrium atmospheric pressure plasma on anti-proliferative activity against chronic chemo-resistant ovarian cancer cells *in vitro* and *in vivo*. PLoS One.

[12] Yan, D. *et al*. Stabilizing the cold plasma-stimulated medium by regulating medium’s composition. *Sci. Rep*. **6** (2016). 10.1038/srep26016 PMC4865954 27172875

[13] Yan D (2014). Controlling plasma stimulated media in cancer treatment application. Appl. Phys. Lett..

[14] Stoffels E, Sakiyama Y, Graves DB (2008). Cold atmospheric plasma: charged species and their interactions with cells and tissues. IEEE Trans. Plasma Sci..

[15] Kong MG (2009). Plasma medicine: an introductory review. New J. Phys..

[16] Kalghatgi S (2011). Effects of non-thermal plasma on mammalian cells. PLoS One.

[17] Tresp H, Hammer MU, Winter J, Weltmann K, Reuter S (2013). Quantitative detection of plasma-generated radicals in liquids by electron paramagnetic resonance spectroscopy. J. Phys. D: Appl. Phys..

[18] Reuter S (2012). From RONS to ROS: tailoring plasma jet treatment of skin cells. IEEE Trans. Plasma Sci..

[19] Evans H (1989). Cytogenetics: overview. Prog. Clin. Biol. Res..

[20] Ames BN, Shigenaga MK, Hagen TM (1993). Oxidants, antioxidants, and the degenerative diseases of aging. Proc. Natl. Acad. Sci. USA.

[21] Szabó C, Ohshima H (1997). DNA Damage Induced by Peroxynitrite: Subsequent Biological Effects. Nitric Oxide.

[22] Yokoyama M, Johkura K, Sato T (2014). Gene expression responses of HeLa cells to chemical species generated by an atmospheric plasma flow. Biochem. Biophys. Res. Commun..

[23] Wende K (2014). Atmospheric pressure plasma jet treatment evokes transient oxidative stress in HaCaT keratinocytes and influences cell physiology. Cell Biol. Int..

[24] Kurita H, Miyachika S, Yasuda H, Takashima K, Mizuno A (2015). Use of molecular beacons for the rapid analysis of DNA damage induced by exposure to an atmospheric pressure plasma jet. Appl. Phys. Lett..

[25] Antoniu A, Nakajima T, Kurita H, Mizuno A (2014). Safety evaluation of nonthermal atmospheric pressure plasma liquid treatment: Single DNA molecule-based method. J. Electrostat..

[26] Wende K (2016). Risk assessment of a cold argon plasma jet in respect to its mutagenicity. Mutat. Res. Genet. Toxicol. Environ. Mutagen..

[27] Kaushik NK, Uhm H, Choi EH (2012). Micronucleus formation induced by dielectric barrier discharge plasma exposure in brain cancer cells. Appl. Phys. Lett..

[28] Kieft IE, Darios D, Roks AJ, Stoffels E (2005). Plasma treatment of mammalian vascular cells: a quantitative description. IEEE Trans. Plasma Sci..

[29] Alimba CG, Dhillon V, Bakare AA, Fenech M (2016). Genotoxicity and cytotoxicity of chromium, copper, manganese and lead, and their mixture in WIL2-NS human B lymphoblastoid cells is enhanced by folate depletion. Mutat. Res. Genet. Toxicol. Environ. Mutagen..

[30] Umegaki K, Fenech M (2000). Cytokinesis-block micronucleus assay in WIL2-NS cells: a sensitive system to detect chromosomal damage induced by reactive oxygen species and activated human neutrophils. Mutagenesis.

[31] Fenech M, Morley AA (1986). Cytokinesis-block micronucleus method in human lymphocytes: effect of *in vivo* ageing and low dose X-irradiation. Mutat. Res. Genet. Toxicol. Environ. Mutagen..

[32] Xing X (2015). Application of human cell transformation assay on assessment of carcinogenic potential of river organic pollutants. Toxicol. Res..

[33] Nersesyan, A. *et al*. Use of the lymphocyte cytokinesis-block micronucleus assay in occupational biomonitoring of genome damage caused by *in vivo* exposure to chemical genotoxins: Past, present and future. *Mutat. Res. Rev. Mutat. Res*. (2016). 10.1016/j.mrrev.2016.05.003 27894679

[34] Sharif R, Thomas P, Zalewski P, Graham RD, Fenech M (2011). The effect of zinc sulphate and zinc carnosine on genome stability and cytotoxicity in the WIL2-NS human lymphoblastoid cell line. Mutat. Res. Genet. Toxicol. Environ. Mutagen..

[35] OECD. Test No. 487: *In Vitro* Mammalian Cell Micronucleus Test. (OECD Publishing).

[36] IAEA. Cytogenetic Dosimetry: Applications in Preparedness for and Response to Radiation Emergencies. (IAEA, 2011).

[37] Levy JA, Virolainen M, Defendi V (1968). Human lymphoblastoid lines from lymph node and spleen. Cancer.

[38] Zighelboim J, Lichtenstein A (1980). Peripheral blood lymphocyte receptors for B-lymphoblastoid cell lines (B-LCL). Blood.

[39] Levy JA, Buell DN, Creech C, Hirshaut Y, Silverberg H (1971). Further Characterization of the WI-L1 and WI-L2 Lymphoblastoid Lines. J. Natl. Cancer Inst..

[40] Gutiérrez-Enrí S, Hall J (2003). Use of the cytokinesis-block micronucleus assay to measure radiation-induced chromosome damage in lymphoblastoid cell lines. Mutat. Res. Genet. Toxicol. Environ. Mutagen..

[41] Sharif R, Thomas P, Zalewski P, Fenech M (2015). Zinc supplementation influences genomic stability biomarkers, antioxidant activity, and zinc transporter genes in an elderly Australian population with low zinc status. ‎Mol. Nutr. Food Res..

[42] Collection, A. T. C. Catalogue of Cell Lines and Hybridomas. American Type Culture Collection 2000).

[43] Fenech M (2007). Cytokinesis-block micronucleus cytome assay. Nat. Protoc..

[44] Fenech M (2002). Chromosomal biomarkers of genomic instability relevant to cancer. Drug Discov Today.

[45] Fenech M, Crott JW (2002). Micronuclei, nucleoplasmic bridges and nuclear buds induced in folic acid deficient human lymphocytes—evidence for breakage–fusion-bridge cycles in the cytokinesis-block micronucleus assay. Mutat. Res. Fund. Mol. Mech. Mut..

[46] Traylor MJ (2011). Long-term antibacterial efficacy of air plasma-activated water. J. Phys. D: Appl. Phys..

[47] Fenech M (2011). The HUMN and HUMNxL international collaboration projects on human micronucleus assays in lymphocytes and buccal cells—past, present and future. Mutagenesis.

[48] Thomas P, Umegaki K, Fenech M (2003). Nucleoplasmic bridges are a sensitive measure of chromosome rearrangement in the cytokinesis-block micronucleus assay. Mutagenesis.

[49] Rosin MP, Anwar WA, Ward AJ (1994). Inflammation, chromosomal instability, and cancer: the schistosomiasis model. Cancer Res..

[50] Weiss SJ (1980). The role of superoxide in the destruction of erythrocyte targets by human neutrophils. J. Biol. Chem..

[51] Henzler T, Steudle E (2000). Transport and metabolic degradation of hydrogen peroxide in Chara corallina: model calculations and measurements with the pressure probe suggest transport of H(2)O(2) across water channels. J. Exp. Bot..

[52] Yan D (2017). The role of aquaporins in the anti-glioblastoma capacity of the cold plasma-stimulated medium. J. Phys. D: Appl. Phys..

[53] Jaruga P, Dizdaroglu M (1996). Repair of products of oxidative DNA base damage in human cells. Nucleic Acids Res..

[54] Henle ES, Linn S (1997). Formation, prevention, and repair of DNA damage by iron/hydrogen peroxide. J. Biol. Chem..

[55] Fenech M (2000). The *in vitro* micronucleus technique. Mutat. Res. Fund. Mol. Mech. Mut..

[56] Lee SL, Thomas P, Fenech M (2015). Genome instability biomarkers and blood micronutrient risk profiles associated with mild cognitive impairment and Alzheimer’s disease. Mutat. Res. Fund. Mol. Mech. Mut..

[57] Frei B, Stocker R, Ames BN (1988). Antioxidant defenses and lipid peroxidation in human blood plasma. Proc. Natl. Acad. Sci. USA.

[58] Vandamme M (2012). ROS implication in a new antitumor strategy based on non‐thermal plasma. International Journal of Cancer.

[59] Oh J-S (2016). How to assess the plasma delivery of RONS into tissue fluid and tissue. J. Phys. D: Appl. Phys..

[60] Oh J-S (2016). How plasma induced oxidation, oxygenation, and de-oxygenation influences viability of skin cells. Appl. Phys. Lett..

[61] Robinson DE (2016). Plasma Polymer and Biomolecule Modification of 3D Scaffolds for Tissue Engineering. Plasma Process. Polym..

